# Impact on clinical outcomes from transcatheter closure of the Fontan fenestration: A systematic review and meta-analysis

**DOI:** 10.3389/fped.2022.915045

**Published:** 2022-10-04

**Authors:** Christopher E. Greenleaf, Zhia Ning Lim, Wen Li, Damien J. LaPar, Jorge D. Salazar, Antonio F. Corno

**Affiliations:** ^1^Pediatric and Congenital Cardiac Surgery, Children’s Heart Institute, Memorial Hermann Children’s Hospital, UTHealth, McGovern Medical School, Houston, TX, United States; ^2^University College of London (UCL) Great Ormond Street Institute of Child Health, University College London, London, United Kingdom; ^3^Division of Clinical and Translational Sciences, Department of Internal Medicine, UTHealth, McGovern Medical School, Houston, TX, United States

**Keywords:** congenital heart defects, congenital heart surgery, fenestration, Fontan circulation, meta-analysis, systematic literature review, univentricular hearts

## Abstract

**Background:**

Meta-analysis of the impact on clinical outcome from transcatheter closure of Fontan fenestration.

**Methods:**

Cochrane, Embase, MEDLINE, and Open-Gray were searched. Parameters such as changes in oxygen saturation, cavo-pulmonary pressure, maximum heart rate during exercise, exercise duration, and oxygen saturation after fenestration closure were pooled and statistical analysis performed.

**Results:**

Among 922 publications, 12 retrospective observational studies were included. The included studies involved 610 patients, of which 552 patients (90.5%) had a fenestration. Of those patients, 505 patients (91.5%) underwent attempt at trans-catheter closure. When it could be estimated, the pooled overall mean age at trans-catheter fenestration closure was 6.6 ± 7.4 years, and the mean follow-up time was 34.4 ± 10.7 months. There were 32 minor (6.3%) and 20 major (4.0%) complications during or after trans-catheter Fontan fenestration closure. The forest plots demonstrate that following fenestration closure, there was a significant increase in the mean arterial oxygen saturation of 7.9% (95% CI 6.4–9.4%, *p* < 0.01). There was also a significant increase in the mean cavo-pulmonary pressure of 1.4 mmHg (95% CI 1.0–1.8 mmHg, *p* < 0.01) following fenestration closure. The exercise parameters reported in 3 studies also favored closing the fenestration as well, yet the exercise duration increase of 1.7 min (95% CI 0.7–2.8 min, *p* < 0.01) after fenestration closure is probably clinically insignificant.

**Conclusion:**

Late closure of a Fontan fenestration has the impact of improving resting oxygen saturation, exercise oxygen saturation, and a modest improvement of exercise duration. These clinical benefits, however, may be at the expense of tolerating slightly higher cavo-pulmonary mean pressures.

## Introduction

In the 50 years following the introduction of the procedure named after Francis Fontan (1), the indications as well as the surgical techniques have substantially evolved. The Fontan operation, which may be performed with various surgical techniques, is the final stage of surgical palliation for functionally univentricular hearts (1–20). A surgically created fenestration (or connection) between the Fontan circuit and the atrial cavity to reduce excessively elevated systemic venous pressures and to improve cardiac output by increasing the filling of the single ventricle may be beneficial in the immediate post-operative period (21–24). To date, the only available prospective randomized study examining the impact of Fontan fenestration demonstrated a reduction in hospital and intensive care unit length of stays with a fenestration (25), even if other studies reported data on this issue (26–28). These potential benefits, however, may be realized at the expense of lower systemic oxygenation, an increased risk of systemic embolism, and the possible need for catheter-based fenestration closure later in life (29, 30).

The long-term management of Fontan fenestrations remains controversial. First, catheter-based Fontan fenestration closure is not without risk, and the concerns related to a closed or absent fenestration are still present long after the postoperative period. Second, long-term systemic thromboembolic risk with an open fenestration has to be balanced against the Fontan paradox of relative systemic venous hypertension and pulmonary arterial hypotension. Third, after Fontan failure develops, the gold standard of treatment is heart transplant. As donor hearts for transplantation remain a scarce resource, other palliative strategies including trans-catheter Fontan fenestration enlargement or creation have been attempted. These attempts were made to improve quality of life, reduce failure symptoms, and possibly improve waitlist safety.

Unfortunately, the overwhelming majority of published literature examining outcomes after transcatheter closure of Fontan fenestrations are observational in nature and underpowered to detect important differences. Heterogenous indications between centers and unclear indications within centers, prevent concrete conclusions of management of the Fontan fenestration in the catheterization lab. To address these limitations, the present systematic review and meta-analysis was conducted to assess the consequences of trans-catheter Fontan fenestration closure on clinical outcomes.

## Materials and methods

This analysis was registered on Prospero (CRD42019139395) on August 21st, 2019. The study was conducted in accordance with the Meta-Analysis of Observational Studies in Epidemiology Guidelines (31). The manuscript was structured in accordance with Systematic Reviews and Meta-Analyses (PRISMA) guidelines and recommendations (32). All reviewed literature was assessed using the Cochrane tools, which cover six domains of bias: selection bias, performance bias, detection bias, attrition bias, reporting bias, and other bias (33). The PICO question for this systematic review and meta-analysis is in fenestrated Fontan patients, what is the change in clinical outcomes from baseline or fenestrations that are left open associated with transcatheter fenestration closure?

### Search strategy

**FIGURE 1:**
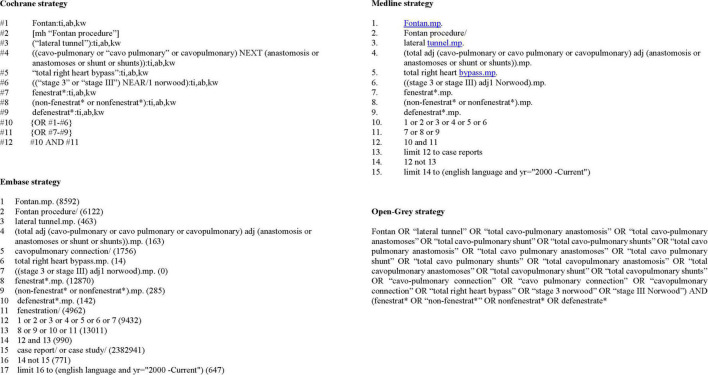
Literature search strategy.

A systematic search was conducted on Cochrane, Embase and MEDLINE. Search terms are provided in Figure 1. Moreover, to avoid losing any related publications, an Open-Gray search was conducted. Related journals and reference lists of identified articles were cross-checked for other relevant studies of interest. Retrospective and prospective observational or randomized controlled trials from the year 200, written in English, reporting the pre-determined outcomes including children or adults undergoing catheter-based intervention on a Fontan fenestration in human subjects were included. Exclusion criteria were case reports, non-original articles, systematic reviews, meta-analyses, non-published works, studies not describing any of the pre-determined outcome measures, or studies that included > 20% of spontaneous fenestration closures. The search was conducted with the assistance of two experienced librarians.

Two independent reviewers (Z.N.L. and A.F.C.) screened all identified studies. In case of multiple publications with sample overlap, the most recent report was included. In each article, the criteria for inclusion and exclusion were independently evaluated by the two reviewers to verify the correctness of selection. In the case of disagreement between reviewers, a consensus was agreed upon. In multiple studies with overlapping study populations, the study with the greatest overall follow-up was included. The first author and/or the corresponding author of three of the included studies were contacted to clarify reported data, particularly regarding the size of the fenestration at the time of surgery.

### Data extraction

Study design, year of Fontan surgery, surgical type of Fontan, fenestration use, and clinical follow-up were documented. The baseline demographics were extracted from the individual studies. The outcomes extracted included early or late mortality, Fontan takedown, heart transplantation, stroke, thromboembolism, or peri-interventional complications. Per-interventional changes in vital signs and changes in hemodynamic parameters including cardiac index, exercise duration, minute ventilation, maximal oxygen consumption, peak exercise oxygen pulse, and ventilatory anaerobic threshold were extracted. Additional clinical outcomes of interest, including protein-losing enteropathy, plastic bronchitis, and arrhythmias were similarly recorded. Complications were deemed major if the grade was > 2 or minor if the grade was ≤ 2 on the Clavien-Dindo classification system (34).

### Statistical analysis

A meta-analysis was performed to compare the outcomes before and after catheter-based Fontan fenestration closure. Outcomes were compared before and after fenestration enlargement or creation for Fontan failure. The mean differences and the corresponding 95% confidence intervals (CI) were estimated using a random effects meta-analysis model, which accounts for variability induced by between-study heterogeneity. Cochran’s Q statistic and *I*^2^ index was used to quantify and test heterogeneity between studies (35). All statistical analyses were performed using R software version 3.6.3 (36) and the meta-package (37). Probabilities with *P*-value < 0.05 were considered statistically significant, and all statistical tests were two-sided.

## Results

**FIGURE 2:**
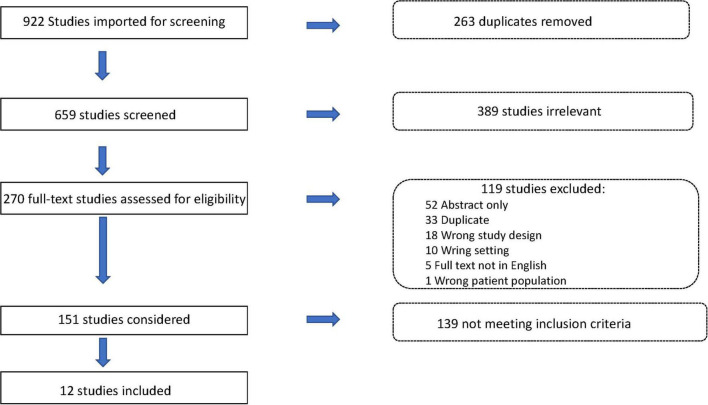
Flowchart of articles screening.

A complete literature search resulted in 922 candidate publications, of which 263 were removed as duplicates. Of the remaining 659 articles, 389 were excluded due to irrelevance. Of the remaining 151 studies, 12 articles met final inclusion criteria (Figure 2). References are represented together with the baseline characteristics of all individual studies. Of the included studies, only 1 study (8%) included all of their Fontan patients done at their center. The rest of the studies used a selected cohort of their overall Fontan population to describe in their study. The included studies involved 610 patients, of which 552 patients (90.5%) had a fenestration. Of those patients, 505 patients (91.5%) underwent attempt at trans-catheter closure.

**FIGURE 3:**
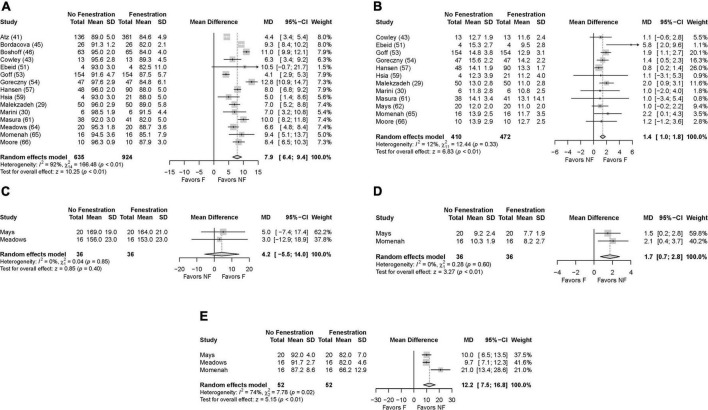
Forest plots after closure of the fenestration. **(A)** Oxygen saturation, **(B)** pulmonary artery pressure, **(C)** exercise heart rate, **(D)** exercise duration, **(E)** exercise oxygen saturation.

When it could be estimated, the pooled overall mean age at trans-catheter fenestration closure was 6.6 ± 7.4 years, and the mean follow-up time was 34.4 ± 10.7 months (Table 1). There were 32 minor (6.3%) and 20 major (4.0%) complications during or after trans-catheter Fontan fenestration closure. The 3 most common minor complications were 10 patients (2.0%) who needed new medications for a diagnosis of heart failure, 8 patients (1.6%) with an arrhythmia, and 4 patients (0.8%) with a vascular access site bleed. The 3 most common major complications were 6 patients (1.2%) who needed device retrieval for failure or malposition, 2 long-term mortalities (0.4%), and 1 early death (0.2%). Forest plots were constructed containing the individual and pooled mean differences for the oxygen saturation, cavo-pulmonary pressure, maximum heart rate during exercise, and exercise duration and oxygen saturation after fenestration closure are presented in Figures 3A–E. The forest plots demonstrate that following fenestration closure, there was a significant increase in the mean arterial oxygen saturation of 7.9% (95% CI 6.4–9.4%, *p* < 0.01) (Figure 3A). There was also a significant increase in the mean cavo-pulmonary pressure of 1.4 mmHg (95% CI 1.0–1.8 mmHg, *p* < 0.01) (Figure 3B) following fenestration closure. The exercise parameters reported in 3 studies also favored closing the fenestration as well (Figures 3C–E), yet the exercise duration increase of 1.7 min (95% CI 0.7–2.8 min, *p* < 0.01) (Figure 3D) after fenestration closure is probably clinically insignificant.

**TABLE 1:** Patients who underwent late closure of the fenestration (> 30 days after Fontan procedure).

Study	Year of publication	Inclusion period	Type of study	Fontan patients, N	Patients with a fene-stration, N	Fene-stration size, mm	Mean age in years at trans-catheter closure ± SD, or median (range)	Trans-catheter fene- strations closure, N	% Fene-strations closed in the cath lab, %	Minor complication (Clavien-Dindo grade = 2)	Manor complication (Clavien-Dindo grade >2)	Early mortality	Late mortality	Total follow- up after closure
Bordacovae t al. 44	2007	2002–2006	Retrospective case series	26	26	Mean 3.5 (3–4)	10.4 ± 0.8	26	100	0	1	0		NA
Boshoff et al. 45	2010	2001–2009	Retrospective cohort study	68	65	Median 5 (4–6)	6.4 ± 2.9	63	96.9	5	7	0		NA
Cowley et al. 48	2000	1998–1999	Retrospective case series	13	13	4	4.6 ± 4.8	13	100	2	2	0		10.4 ± 4.6 months
Goff et al. 52	2000	1989–1999	Cross sectional study	154	154	4	4.2 ± 11.8	154	100	7	5	0	2	Median3.4 years(0.4–10.3 years)
Goreczny et al. 53	2017	2000–2014	Case control study	102	47	5.1 ± 1.2	Median 6.1 (3.9–10.6)	47	100	7	3	0	0	Median19.6(8–33.5)months
Hansen et al. 56	2012	1996–2010	Retrospective cohort study	90	90	>4	Median 4.4 (1–14.2)	48	53.3	NA	NA	NA	NA	66 ±36.3months
Malekzadeh et al. 29	2015	2005–2012	Retrospective case series	50	50	5, 6, 7	7.8 ± 3.8	50	100	6	2	1	0	49 months
Masura et al. 60	2008	1997–2007	Retrospective case series	41	41	NA	Median 8 (2.5–26)	38	92.7	3	0		0	Median12(0.1–10 years
Mays et al. 61	2008	NA	Retrospective observational study	20	20	NA	11.4± 5.5	20	100	2	0			NA
Meadows et al. 63	2008	2005–2007	Prospective observational study	20	20	4	13.8 ± 10.4	20	100	NA	NA	0	0	12 months
Momenahet al. 65	2007	NA	Retrospective case series	16	16	4, 5, 6	Median 10.3 (6–13)	16	100	NA	NA	0	0	NA
Moore et al. 66	2000	1998–1999	Retrospective case series	10	10	4, 5, 6	7.0 ± 4.1	10	100	0	0	0	0	6 months

## Discussion

The present systematic review and meta-analysis suggests that trans-catheter closure of a Fontan fenestration is associated with improved resting and exercise oxygenation, lower maximal heart rate during exercise, and longer exercise duration (Figures 3A,C–E), even if this is at the expense of slightly higher pulmonary artery mean pressures (Figure 3B). This analysis demonstrates that the immediate results of Fontan fenestration closure are fairly consistent. There is a systemic oxygen saturation mean increase of 7.9% (range 4.1–12.8%, Figure 3A). The pulmonary pressure slightly increases by 1.4 mm Hg (range 0.8–5.8 mm Hg, Figure 3B). Exercise tolerance does increase, but this length is short and was only tested in 2 studies (Figure 3C).

A fenestration eases the transition to the Fontan circulation for patients by providing a consistent source of systemic ventricular preload. Secondary benefits include decreased postoperative pleural effusions and hospital length of stay (25, 38). Fenestrating most Fontans has become commonplace in the modern era of Fontan management.

### Different institutional practices toward the management of the Fontan fenestration

There is no concrete guidance on what should be done for the fenestration over the medium or the long-term. Because of this, there is dramatic heterogeneity fenestration management amongst centers. Because of the theoretic risk of thromboembolism and detrimental effects of prolonged cyanosis, some centers routinely close all fenestrations in the catheterization lab at about 12 months postoperatively (39). Patients who had spontaneous closure of their fenestration probably did so because of low trans-pulmonary pressures and resultant minimal relative flow across their fenestrations. This patient population, who can make up to about 40% of fenestrated Fontan patients, represent a low risk for Fontan failure (40). The long-term answer surrounding the question of what to do for the other 60% of patients was not clear. A persistent fenestration may be a surrogate for physiologic intolerance of the Fontan circulation, and this difference may not be readily apparent by pre-Fontan hemodynamic parameters (41–75). This has led some centers to either shift from routine closure to not closing any fenestrations (76) or to be very selective in which patients are referred for transcatheter closure (54, 62). For instance, McCrossan and Walsh (62). Will refer patients for trans-catheter Fontan fenestration closure if there is persistent hypoxia, satisfactory ventricular function, and absence of significant Fontan circuit obstruction.

### Contraindications for fenestration closure

After the patient is referred to the catheterization lab, the interventional cardiologist may find that the patient is not appropriate for closure. Many centers use the parameters set forth by the group at Boston Children’s Hospital in 1995 (77). At the time of catheterization and after measuring baseline features, the fenestration is then test occluded for 10 min. If the right atrial pressure exceeded 18 mmHg, or the arteriovenous difference in oxygen saturation increased by > 33%, or the right atrial saturation was < 40%, then the patient was considered to have an unfavorable response to test occlusion ad the fenestration was left open. Similarly, Goreczny et al. will do the 10-min test occlusion and will avoid permanent fenestration closure if the systemic venous pressure is above 18 mmHg or if there is an increase of 5 mmHg after balloon occlusion (53). At the Children’s Hospital of Michigan closure is deferred after balloon occlusion if the Fontan pressure is ≥ 20 mmHg, the Fontan pressure increases by 4 mmHg or more, if there is a decrease in cardiac output by ≥ 50%, or a decrease in systemic oxygen transport of ≥ 46% (78).

These strict criteria during test occlusion may identify patients that would not tolerate fenestration closure, but it may be that this provocative measure is sensitive but not specific for those patients that may not have the proper hemodynamics but would tolerate closure anyway. Ozawa et al. studied the long-term outcomes after fenestration closure in patients at risk for Fontan failure. They compared high-risk Fontan patients, as defined by pre-Fontan mean pulmonary arterial pressure ≥ 15 mmHg or systemic atrio-ventricular valve regurgitation ≥ moderate, compared to a standard risk group and a group whose fenestration had closed spontaneously. Protein-losing enteropathy-free survival rates did not differ between groups (*p* = 0.72). This was at the expense, in the high-risk group, of persistent cyanosis from veno-venous collaterals and lower peak oxygen consumption (*p* = 0.019) and lower anaerobic threshold (*p* = 0.023) compared to the standard risk group (79).

### Exercise capacity

Exercise capacity as described by maximal oxygen consumption (VO_2_ max) has found diametrically opposite conclusions in certain reports. Mays et al. investigated 20 patients before and after Fontan fenestration closure. In their analysis, the VO_2_ max increased to 1.24 ± 0.35 L/min from 1.18 ± 0.46 L/min, *p* < 0.005 (61). Conversely, Meadows and colleagues also investigated 20 patients before and after fenestration closure. In this series, the percent predicted VO_2_ max increased to 74 ± 18.6% from 70.9 ± 18.6%, *P* = NS. In both studies, there was a wide variation in patient age and size (63). To take into account growth-related changes between pre- and post-fenestration closure exercise tests, different measurement endpoints were considered. Such differences may explain why exercise capacity was statistically significant in one study but not the other. Another potential explanation may be a combination of competing interests. One would surmise that increasing systemic arterial saturation would improve VO_2_ max and exercise duration, but this increase in systemic arterial saturation was at the expense of cardiac index and mixed venous oxygen saturation. Furthermore, neither study was able to define the amount of right to left shunting under exercise conditions. In his series Meadows stated that, at rest in the catheterization lab, all patients had no more than trivial shunting at the end of the catheterization, but that does not exclude the possibility that more significant shunting could have occurred with effort (63). The cardiopulmonary response to exercise in the Fontan circulation is complicated by an inability to regulate heart rate or stroke volume in response to exercise, systemic venous flow dynamics, and the compound contribution of ventilation on the Fontan circuit.

### Long-term consequences of an open or closed fenestration

As stated earlier, the Fontan fenestration is associated with well documented early postoperative benefits. Studies identifying long-term benefits are sparse. Atz et al. showed a significant increase in the rate of fenestration across multiple centers between 1987 and 2002. After adjusting for era, patient age, and year of Fontan, this large, multi-center cross-sectional study found few associations between a persistent fenestration and negative long-term outcomes (40). There was a greater number of non-fenestrations associated catheter re-interventions, most commonly for coiling of systemic venous and aorticopulmonary collaterals. Unsurprisingly, the resting oxygen saturation was lower with a fenestration in the long-term, but they found no difference in the number of long-term re-interventions, incidence of protein-losing enteropathy or arrhythmias. The Australian and New Zealand Fontan registry has been a wealth of knowledge in understanding the Fontan outcomes in the modern era (80). In a propensity score-matched analysis they demonstrated that there was no difference in long-term survival (87% vs. 90% at 20 years; *p* = 0.16) or freedom from failure (73% vs. 80% at 20 years; *p* = 0.10) between patients with and without fenestration, respectively. There was more freedom from thromboembolism in the non-fenestrated vs. the fenestrated group (89% vs. 84%; *p* = 0.03) (80). On the other hand, in the study from Children’s Hospital of Michigan, the incidence of a composite outcome of death, transplant, deteriorated heart failure, plastic bronchitis, or protein-losing enteropathy was significantly higher when there was an open fenestration (60% vs. 6%; *p* < 0.01) (78). As mentioned earlier the patients with the open fenestration in this study were a select group of patients who failed to meet proper hemodynamics with test occlusion which may be the difference in the findings between these studies (78).

### Limits of the study

The present review and meta-analysis have select limitations. First, a large number of published articles reported incomplete data. For instance, no suitable data were reported to analyze the impact of complications after closure of the fenestration, such as stroke and/or systemic thrombo-embolism, as well as the occurrence of liver failure due to the increased systemic venous pressure. We therefore limited our presentation of the results to the most relevant information in regard to the long-term outcome of fenestration. Second, data extracted from observational study designs should be interpreted with caution due to the inherent limitations of confounding and a high degree of selection bias. Most importantly, select studies on the management of Fontan fenestration in patients with failed Fontan circulation had inadequate statistical analyses, which limited the ability to rigorously assess this fundamental question in the present analysis. Third, the first two limits are the consequence of the considerable controversy and widely varying institutional practices concerning Fontan fenestrations in respect to indication for establishment as well as management during long-term follow-up.

## Conclusion

Late closure of a Fontan fenestration has the impact of improving resting oxygen saturation, exercise oxygen saturation, and a modest improvement of exercise duration. These clinical benefits, however, may be at the expense of tolerating slightly higher cavo-pulmonary mean pressures. There is a substantial lack of high-quality evidence supporting any therapeutic decision regarding Fontan fenestration, and this is reflected in the difficulties encountered in our literature review and meta-analysis.

## Data availability statement

The original contributions presented in this study are included in the article/Supplementary material, further inquiries can be directed to the corresponding author.

## Author contributions

All authors contributed to the idea and design of the study, preparation, review and approval of the manuscript.
